# Supplementary material for: Genome-Wide Identification of Wheat KNOX Gene Family and Functional Characterization of TaKNOX14-D in Plants
Source: Int J Mol Sci. 2022 Dec 14;23(24):15918. doi: 10.3390/ijms232415918 (PMC9784718; doi:10.3390/ijms232415918)
Supplement: Supplementary file 1 [file ijms-23-15918-s001.zip › Supplementary Figure.pdf]

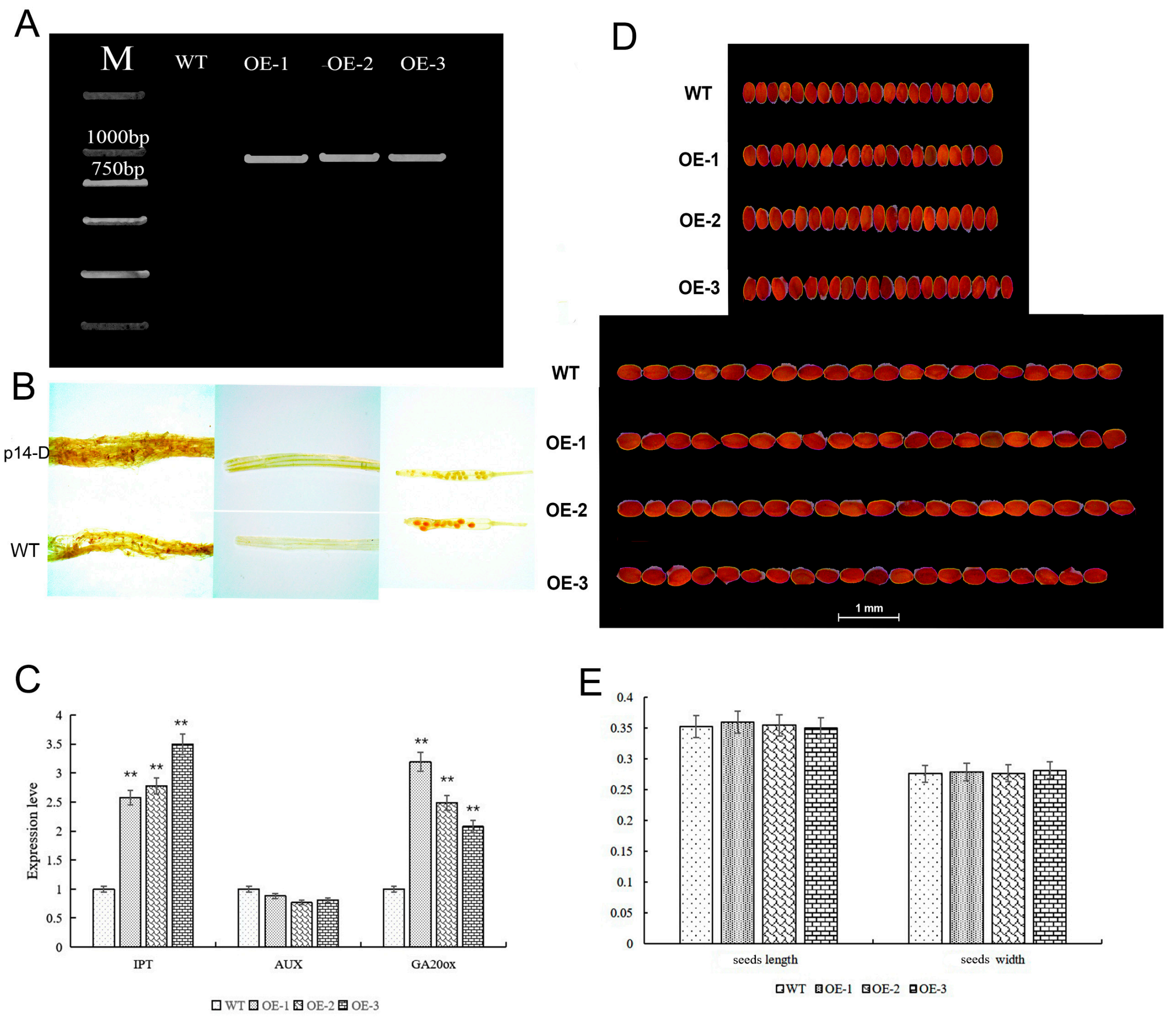

Figure S1. Phenotypes of WT and three transgenic Arabidopsis overexpressing TaKNOX14-D. (A): TaKNOX14-D gene was detected in transgenic lines. M: marker 2000, 1000bp and 750bp represented the length of sequence, the sequence length of TaKNOX14-D was 930bp. (B): GUS staining of root, leaf, stem and pod. (C): The expression pattern of IPT, AUX and Ga20ox gene in four Arabidopsis lines. (D) and (E): Seed length and width after mechanical damage, bar=1mm. wenty seeds were randomly chosen and the seed width/length were counted. *, P < 0.05; **, P < 0.01 (Student's t-test)
